# KAP1 targets actively transcribed genomic loci to exert pleomorphic effects on RNA polymerase II activity

**DOI:** 10.1098/rstb.2019.0334

**Published:** 2020-02-10

**Authors:** Annamaria Kauzlaric, Suk Min Jang, Mehdi Morchikh, Marco Cassano, Evarist Planet, Monsef Benkirane, Didier Trono

**Affiliations:** 1 School of Life Sciences, Ecole Polytechnique Fédérale de Lausanne (EPFL), 1015 Lausanne, Switzerland; 2 Laboratory of Molecular Virology, Institute of Human Genetics, CNRS UPR1142, MGX-Montpellier GenomiX, 141 rue de la Cardonille, 34396 Montpellier, France; 3 Institute of Molecular Genetics of Montpellier, CNRS, Université de Montpellier, 34090 Montpellier, France

**Keywords:** KAP1/TRIM28, epigenetics, histone modification, transcription, RNA polymerase II, mass spectrometry

## Abstract

KAP1 (KRAB-associated protein 1) is best known as a co-repressor responsible for inducing heterochromatin formation, notably at transposable elements. However, it has also been observed to bind the transcription start site of actively expressed genes. To address this paradox, we characterized the protein interactome of KAP1 in the human K562 erythro-leukaemia cell line. We found that the regulator can associate with a wide range of nucleic acid binding proteins, nucleosome remodellers, chromatin modifiers and other transcription modulators. We further determined that KAP1 is recruited at actively transcribed polymerase II promoters, where its depletion resulted in pleomorphic effects, whether expression of these genes was normally constitutive or inducible, consistent with the breadth of possible KAP1 interactors.

This article is part of a discussion meeting issue ‘Crossroads between transposons and gene regulation’.

## 1. Introduction

KAP1/TRIM28 is a master regulator critical to processes such as stem cell self-renewal and DNA-damage response [1–6]. Its best-described function thus far is the binding through sequence-specific targeting Krüppel-associated box domain-containing-zinc finger proteins (KZFPs) to transposable elements (TEs), which leads to the transcriptional silencing of these genetic units [7–12]. At these sites, KAP1 acts as a scaffold protein for the formation of a heterochromatin-inducing machinery comprising HP1 (heterochromatin protein 1), the histone methyl-transferase SETDB1, the nucleosome remodelling and deacetylase (NuRD) complex and the histone demethylase KDM1A [9,13–17]. Yet a growing number of studies additionally describe a diametrically different pattern of genomic recruitment for KAP1, with its accumulation at promoters of actively transcribed genes, independently of KZFPs or any silencing complex [1,13,18–22]. The mediators and associated effectors, as well as functional consequences of KAP1 recruitment at these loci, are still debated. A model was proposed whereby KAP1 directly bound the DNA fibre and acted as an RNA polymerases II (PolII) stalling factor at gene promoters, where upon appropriate stimuli, it underwent phosphorylation of serine 824 (pS824), which resulted in releasing paused PolII, hence allowing or enhancing transcription of the underlying gene [19]. Another study centred on KAP1 recruitment at gene promoters also found it to correlate with paused PolII genome-wide, and while the molecular mechanisms of its recruitment remained unexplored, there was evidence of KAP1-mediated transcriptional control for inducible genes [21]. At promoters of these genes, KAP1, together with the 7SK and small nuclear ribonucleoprotein (snRNP) complex, appeared to function as a dynamic supplier of inactive positive transcription elongation factor b (P-TEFb), facilitating the transition from the initiation to the elongation phase of PolII-mediated transcription. While some of the findings reported in these two studies coincided, functional data strikingly diverged, since in the former KAP1 removal resulted in increased transcription of KAP1-targeted inducible genes, whereas in the latter, these genes were then repressed, while the expression of most of their housekeeping counterparts was unaltered.

Here, we present proteomic and functional data indicating that KAP1 can associate with a wide range of nucleic acid binding proteins, nucleosome remodellers and other transcriptional modulators, that it can associate with promoters recognized by PolII, and that its impact at these genetic loci is diverse, as predicted from the breadth of its protein interactome.

## 2. Results

### (a) The interaction network of KAP1 is enriched in nucleic acid binding proteins

**Figure 1.**
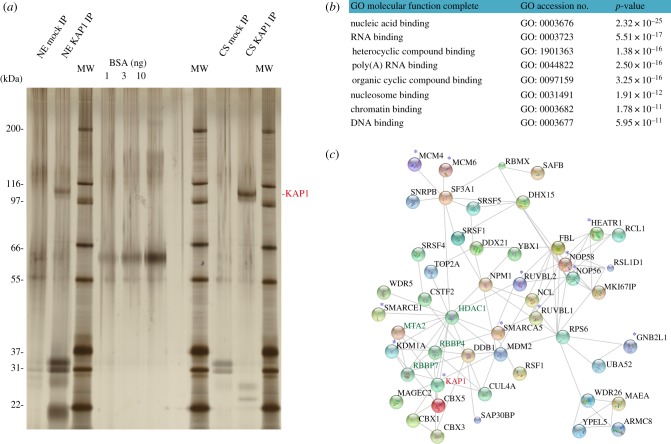
(*a*) Silver-stained SDS-PAGE gel of KAP1 interactome obtained by pull-down of the tagged version of the protein in K562 cells. Lanes are loaded with, from left to right: nuclear extracts (NE) of control (mock) IP, namely the corresponding extracts purified from the control cell line not expressing the tagged version of KAP1, and KAP1 IP; molecular weight (MW) ladder; 1, 3, 10 ng of bovine serum albumin (BSA); MW ladder; chromatin-soluble (CS) mock- and KAP1 IP; MW ladder. (*b*) Gene ontology (GO) analysis of KAP1 interactome, merging the interacting proteins purified from both nuclear and chromatin fractions isolated from K562 cells expressing the tagged version of KAP1. (*c*) Vector representation of KAP1 interactors obtained by pull-down of the tagged version of the protein in K562 cells. Proteins with a total peptide-coverage of 5% or higher, either in the chromatin or in the nuclear fraction, were selected. Lines linking the proteins highlight experimentally determined interactions (http://string-db.org). Disconnected nodes were excluded. Bold labels were used for KAP1 interactors experimentally validated through independent studies (https://thebiogrid.org/). Green labels were used for units of the nucleosome remodelling and deacetylase (NuRD) complex. KAP1 interactors previously detected in unfractionated human embryonic stem cells and K562 cells (threshold *p-*value < 0.01) [23] are indicated with blue asterisks.

We previously examined the interactome of KAP1 by immunoprecipitating the endogenous protein in whole-cell extracts of human embryonic stem and K562 erythro-leukaemic cells. While this approach revealed KAP1 association with proteins critical for DNA replication, such as PCNA, RPA1 and subunits of the MCM (MiniChromosome Maintenance) complex, it did not lend itself to the purification of sufficient amounts of material to allow cell fractionation. We thus turned to immunoprecipitation (IP) of a doubly tagged form of KAP1 in K562 cells, extracting the chromatin from the nuclear fraction and subjecting the products to mass spectrometry (MS/MS) analysis. This tandem affinity-purification, where the sequential use of two high-affinity antibodies increased specificity, revealed higher amounts of KAP1 in chromatin extracts, indicating that in the nucleus, this protein is mainly associated with DNA (figure 1*a* and electronic supplementary material, figure S1A). Fractionation of precipitated complexes revealed a single elution peak extending over the first seven of 25 fractions of a linear 15 to 35% (v/v) glycerol gradient in both nuclear and chromatin extracts. Although not informative of the number of distinct KAP1-associated complexes, it suggests that these are of similar and relatively small molecular weight (MW), with their migration possibly affected by association with nucleic acids (electronic supplementary material, figure S1B). The very top categories of a gene ontology search based on molecular functions of KAP1 interactors included RNA-binding proteins, in addition to DNA- and chromatin-associated proteins (figure 1*b*). Accordingly, a somewhat diffuse band of apparent MW below 20 kDa, detected on the silver stained gel in the total nuclear but not the nuclease-treated chromatin soluble (CS) fraction, strongly suggested that nucleic acids were co-immunoprecipitated with KAP1 (figure 1*a*). Known partners of KAP1, such as components of the NuRD complex and HP1 proteins, associated with overexpressed KAP1 in K562 cells (figure 1*c*). These findings were in agreement with previous MS/MS studies performed by our group with the endogenous protein in human embryonic stem cells (hESC), where we found significant enrichment of the same cofactors [23] (electronic supplementary material, figure S1C,E). In addition, proteins part of the DNA replication and DNA repair machineries, such as components of the MCM protein complex, RuvB-like proteins 1 and 2 (RUVBL1, RUVBL2) and DNA-damage binding protein 1, were co-immunoprecipitated with KAP1 in both experimental settings [23] (electronic supplementary material, figure S1D,E and table S1). Finally, we also detected in these complexes a range of nucleosome remodellers, such as components of SWItch/Sucrose Non-Fermentable (SWI/SNF), and of RNA-binding proteins, namely members of the DEAD-box RNA helicases protein families (electronic supplementary material, figure S1D,E). In particular, both components of the remodelling and spacing factor (RSF), RSF1 and SMARCA5, a complex facilitating activator-dependent PolII transcription initiation [24], were co-purified with KAP1. Moreover, in our experimental settings, we detected a strong enrichment of DDX21, a helicase that functions as PolII transcription elongation factor, by facilitating the release of the P-TEFb from the 7SK snRNP complex [25], as well as genome stability guardian [26]. The breadth and functional diversity of the KAP1 interactome strongly suggests that this master regulator fulfils multifaceted functions (figure 1*c*).

### (b) Extensive recruitment of KAP1 to PolII promoters

**Figure 2.**
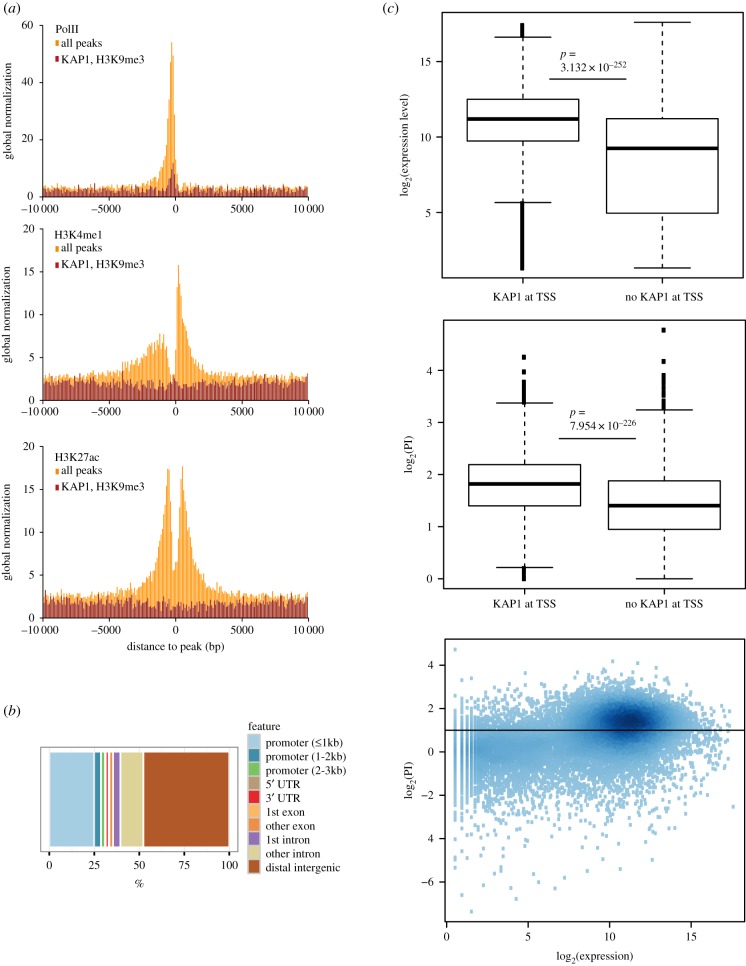
(*a*) Positional correlation between KAP1 binding sites or KAP1 binding sites that overlap H3K9me3-enriched loci and peaks of (top) PolII, (centre) H3K4me1 and (bottom) H3K27ac. A symmetric window of 10 kilobase-pairs (kbp) was considered, and the correlation was normalized by the size of each dataset. (*b*) Annotation of KAP1 peaks over selected genomic features. (*c*) (Top) Boxplot comparison of expression levels and (middle) pausing index (PI) of genes whose promoter region is either bound or not bound by KAP1. (Bottom) Correlation analysis of PI and expression level for detectable genes, with colour shades reflecting different data-point densities (darker colours corresponding to higher densities).

In the light of our MS/MS results and recent findings supporting a putatively direct role of KAP1 in PolII-mediated gene transcription, we sought to explore further correlations between KAP1 genomic recruitment and active transcription. For this, we chose the Hepa 1-6 murine hepatoma cell line, because we had previously documented in these cells an extensive overlap between KAP1 binding and histone marks associated with active gene expression [27]. Of note, we found the general features of KAP1 genomic recruitment to be otherwise similar in these cells to those documented in other settings, whether embryonic or differentiated cells from either human or murine origin [8,10,18,28,29]. Analysis of chromatin IP coupled with deep sequencing (ChIP-Seq) data in Hepa 1-6 cells confirmed a significant overlap of sites targeted by KAP1 with regions enriched in the H3K27ac and H3K4me1 active histone marks, and further revealed its co-localization with PolII at many sites (figure 2*a*). A closer examination indicated that this subset of KAP1-recruiting loci was devoid of the H3K9me3 repressive mark, which otherwise displayed a strong genome-wide correlation with KAP1 peaks at *bona fide* targets of the KAP1–SETDB1 complex, such as TE-derived sequences and the 3′-end of KZFP genes as previously noted in other systems (figure 2*a* and electronic supplementary material, figure S2A,B) [30]. We examined the distribution of KAP1 binding sites over selected gene features and observed a remarkable enrichment at promoters (approximately 25%), in agreement with the strong correlation with PolII-enriched regions (figure 2*b*) and consistent with data previously obtained in other cells [8,18,28,29]. We further observed that KAP1 was preferentially binding the promoters of highly transcribed genes, which in turn were characterized by a high PolII pausing index (PI), defined as the ratio between PolII at the transcription start site (TSS) region and over the gene body, in agreement to previously established methods [31] (figure 2*c*).

### (c) Pleomorphic influences of KAP1 on PolII distribution and transcription

**Figure 3.**
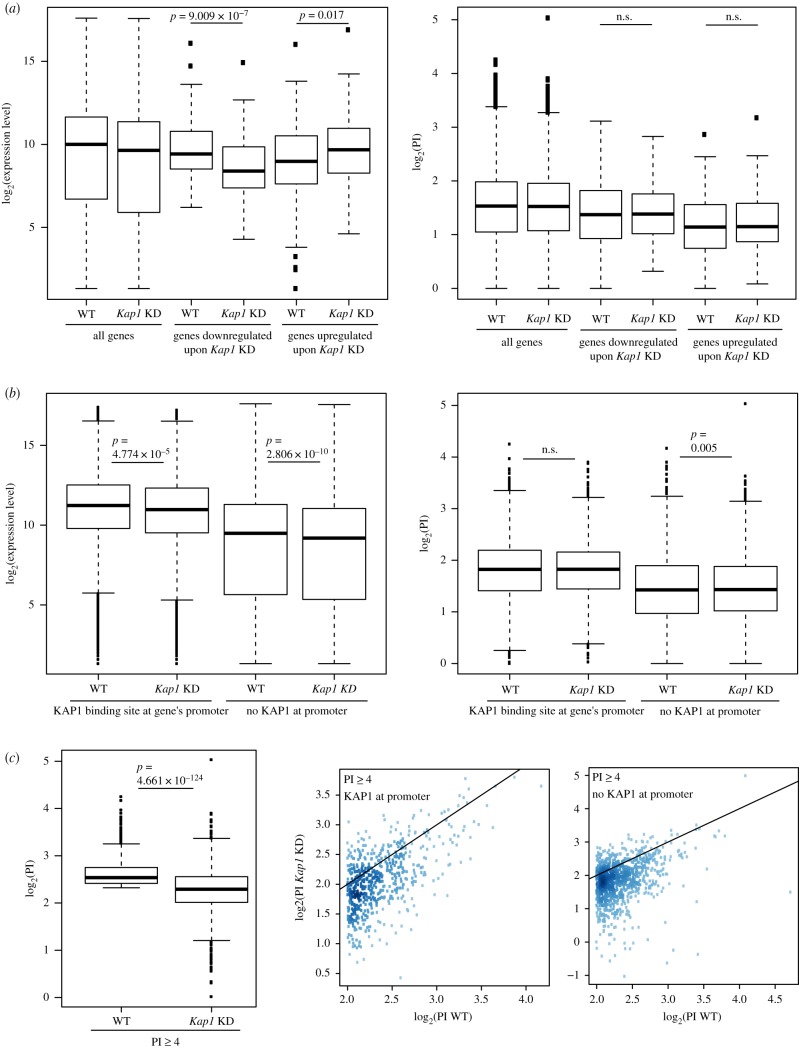
(*a*) Boxplot comparison of (left) expression levels and (right) pausing index (PI) in wild-type (WT) and Kap1 knockdown (KD) cells of the ensemble of detected genes, as compared with genes significantly downregulated or upregulated upon Kap1 KD (‘down’ and ‘up’, respectively). (*b*) Boxplot comparison of (left) expression levels and (right) PI of selected genes in WT and Kap1 KD cells. Detectable genes were separated based on the presence or absence of KAP1 peaks over their promoter regions, as determined by ChIP-Seq in WT settings. The promoter region of a gene was defined by taking its annotated 5′ end position and extending it by 250 nucleotides (nt) in either direction. (*c*) (Left) Boxplot comparing PI in WT and in their Kap1 KD counterpart cells of genes having a PI greater than or equal to 4, as measured in the WT condition (*N* = 1538). (Middle and right) Correlation between PIs measured in WT and Kap1 KD cells for the same group of genes, plotting separately genes bound by KAP1 in their promoter region and genes without a KAP1 peak in that same extremity in WT cells (*N* = 567 and *N* = 971, respectively). The density of data-points in the plot is proportional to the shade of colour (higher density corresponding to darker colours). For all plots, the Mann–Whitney–Wilcoxon test was used to assess the significance.

We then characterized the functional consequences of KAP1 recruitment at these promoters by comparing the transcriptional profiles of control and of *Kap1* knockdown (KD) Hepa 1-6 cells (electronic supplementary material, figure S3A,B). Overall, KAP1 depletion led to limited perturbations of gene expression, with a totals of only 145 downregulated and 134 upregulated PolII genes (figure 3*a*, left). However, we could not correlate either of these changes with specific alterations of PolII pausing index (figure 3*a*, right), determined in both basal and KD conditions. Furthermore, KAP1 binding in the proximity of a TSS was not predictive of the transcriptional deregulation of the corresponding gene in KAP1-depleted cells (figure 3*b*, left). Similarly, the PI of KAP1-targeted promoters did not globally change upon KAP1 removal (figure 3*b*, right and electronic supplementary material, figure S3C). However, when groups of genes with an elevated (≥4) PI at baseline were analysed separately, a significant difference was detected (figure 3*c*, left). This category of genes thus followed a previously reported pattern [32]. Nonetheless, when we separated genes within this group based on whether or not KAP1 was recruited over their promoter region, we observed no deviation from the initial trend (figure 3*c*, right), indicating that for the largest fraction of these genes, PI changes were independent of KAP1 proximal binding.

### (d) Induction of early response genes is variably affected by KAP1

**Figure 4.**
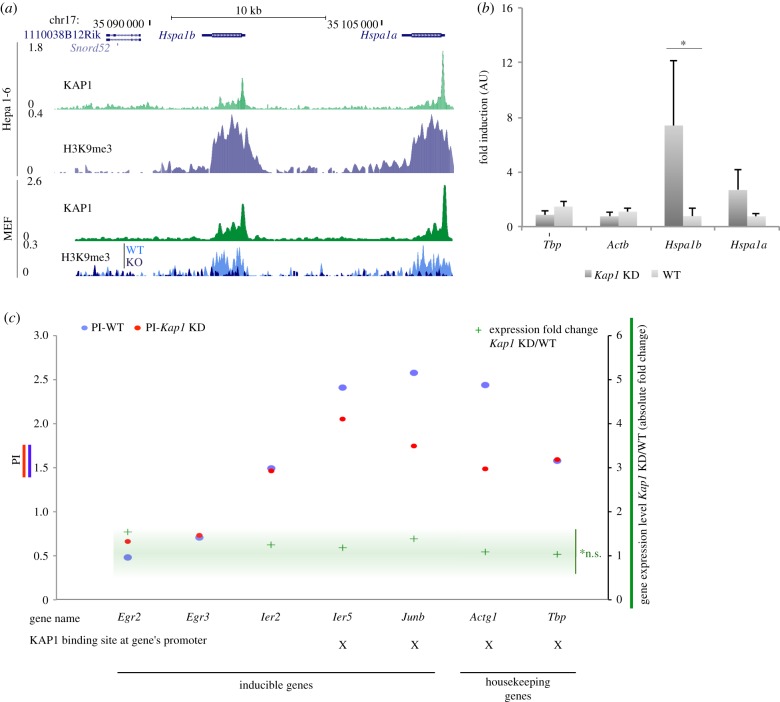
(*a*) Schematic view of the *Hspa1b* and *Hspa1a* loci, displaying, in order from the top: the track of annotated genes, KAP1 and H3K9me3 ChIP-Seq profiles in Hepa 1-6 cells, KAP1 and H3K9me3 ChIP-Seq profiles in MEFs. For the latter, the track of H3K9me3 in Kap1 KO cells was superimposed on to the one in WT cells. (*b*) RT-qPCR analysis of *Tbp, actin β, Hspa1b* and *Hspa1a* gene transcripts in Hepa 1-6 cells WT and Kap1 KD (transduced with an empty and a Kap1-targeting sh-vector, respectively) after heat shock treatment. Expression levels were normalized to actin γ and ratios between the cell line derivatives and their untransduced counterpart are shown. *p-value < 0.05. (*c*) (Left *y*-axis) PI in WT and Kap1 KD Hepa 1-6 cells (in blue and red, respectively) measured for the inducible genes *Egr2, Egr3, Ier2, Ier5* and *Junb*, and the housekeeping genes *Actg1* and *Tbp*. (Right *y*-axis) Absolute value of expression level fold change in Kap1 KD over WT Hepa 1-6 cells measured by mRNA-Seq for the same set of genes. For each gene, we further reported whether its promoter region was bound by KAP1 in WT cells, as determined by ChIP-Seq. n.s., non-significant. For detailed mRNA-Seq analysis methods refer to the electronic supplementary material.

KAP1 was reported to target the promoter and regulate the transcription of inducible genes, including heat shock protein (HSP) genes in the human embryonic kidney cell line HEK293T [19]. We confirmed a marked association of KAP1 with the promoters of *Hspa1a* and *Hspa1b* genes in Hepa 1-6 cells as well as in a distinct cellular system, namely mouse embryonic fibroblasts (MEFs) (figure 4*a*). Remarkably, these genes were strongly enriched in H3K9me3 and completely devoid of the active histone marks H3K27ac and H3K4me1 (figure 4*a* and electronic supplementary material, figure S4A). H3K9me3 at these loci seemed to be KAP1-dependent, as the body of *Hsp* genes lost H3K9me3 in *Kap1* knockout (KO) MEFs (figure 4*a*). We could not confidently assess alterations of the basal expression of *Hspa1a* and *Hspa1b* upon *Kap1* KD, since their transcripts remained at the very limit of detection. Nevertheless, upon heat shock, we could measure a significantly higher increase in their induction in KAP1-depleted cells (figure 4*b* and electronic supplementary material, figure S4B). Immunofluorescence studies further revealed that S824-phosphorylated KAP1 accumulated in the cell nucleus upon heat shock (electronic supplementary material, figure S4C), as previously reported [19]. Another set of inducible genes, including early growth response, immediate early response protein genes and the transcription factor *JunB*, were not deregulated in our datasets upon KAP1 removal at basal level, although several of them displayed mild shifts in PolII distribution (figure 4*c* and electronic supplementary material, figure S4D).

## 3. Discussion

The body of KAP1 interactors reflects its recruitment predominantly to the chromatin fraction of the nucleus. In addition to its known partners in heterochromatin formation and transcriptional silencing, such as HP1 proteins and components of the NURD complex, our analysis of the KAP1 interactome in K562 leukaemic cells identified several factors involved in modulating the structure of euchromatin and the activity of RNA polymerases [24,25,33–37]. This fits with the detection by several studies including ours of KAP1 at the promoters of numerous PolII-transcribed genes.

KAP1 depletion induced pleomorphic effects on the activity of its PolII promoter targets, and our data at least partly invalidated several recently advanced models [19,21]. *Kap1* KD induced a general decrease in PI at genes characterized by a high PI at basal conditions, the vast majority of which was, however, not bound by KAP1 over the promoter region. Moreover, the detected changes in PI were globally of small magnitude and mostly uncoupled from differences in transcriptional levels. In the case of HSP genes, we noted that their epigenetic signature, characterized by strong enrichment in H3K9me3 and lack of active histone modifications, was diametrically different from the one found at other KAP1-bound promoters. Additionally, the high PI of these genes reflected the stalling and inactivity of PolII subunits at their promoters, while the bulk of KAP1-targeted promoters were characterized by high transcriptional activity in addition to high PIs. The expression level of a second group of KAP1-bound inducible genes, comprising transcriptionally active genes that were devoid of the repressive mark H3K9me3, was not shifted by the removal of KAP1 at baseline. Previous investigations illustrating the functional versatility of the regulator, both in its canonical KZFP-mediated TE targeting configuration [38] and in combination with a panel of transcription factors [28,39,40], focused on experimental systems where KAP1 acted either at discrete differentiation stages of selected tissues or in specific signalling pathways. Taken together, these findings indicate that inducible genes represent neither a homogeneous system nor a directly relevant model for studying the roles played by KAP1 at promoters of housekeeping and stably expressed genes, where the regulator must exert influences not detectable in our tissue culture system, or manifested only under exceptional circumstances, as suggested by the viability of mice devoid of KAP1 in the liver or in part of the brain [41,42].

## 4. Experimental procedures

### (a) Cell culture and mouse work

MEFs wild-type (WT) and KO for *Kap1* were cultured and generated as previously described [43] (strain C57BL/6 J). Murine hepatoma Hepa 1-6 and K562 cells were cultured using standard methods.

### (b) Plasmids and lentiviral vectors

For KAP1 KD experiments, pLKO vector encoding shKAP1 and the empty vector as control were used. Forty-eight hours after transduction, infected cells were selected with 2 µg ml^−1^ puromycin in growth medium for an additional 72 h. Lentiviral vector production protocols are available at http://tronolab.epfl.ch and backbones at Addgene (http://www.addgene.org). For the expression of FLAG-HA-KAP1, the human KAP1 partially codon-optimized sequence was cloned in the retroviral pOZ-N vector [44].

### (c) Heat shock

Heat shock was performed by placing the culture dish over a 45°C water bath for 5 min, prior to RNA extraction.

### (d) Immunofluorescence

Cells were fixed in methanol for 5 min at −20°C and labelled with anti-phospho-S824 KAP1 (ab70369) or anti-KAP1 (MAB3662) followed by Alexa488- or Alexa565-conjugated anti-mouse antibody. Nuclei were stained with DAPI (4′,6-diamidino-2-phenylindole). Images were acquired using a 63× lens on a Zeiss Axiovert 200 M microscope.

### (e) RNA purification, RT-PCR (reverse transcriptase PCR) and RNA-Seq

Total RNA was extracted and DNase-I treated using a spin column-based RNA purification kit (Macherey-Nagel). Complementary DNA (cDNA) was prepared with SuperScript II reverse transcriptase (Invitrogen). The sequences of primers used for SYBR green quantitative PCR (qPCR) (Applied Biosystems) are provided in electronic supplementary material, experimental procedures. For the sequencing of mRNA (poly(A)+), 100 bp single-end RNA-Seq libraries were prepared using the Illumina TruSeq mRNA reagents (Illumina). Cluster generation was performed with the resulting libraries using the Illumina TruSeq SR Cluster Kit v4 reagents. Sequencing was performed in 100 bp reads run on an Illumina HiSeq 2500. Further information about the mapping and analysis procedures is provided in electronic supplementary material, experimental procedures.

### (f) ChIP-qPCR and ChIP-Seq

ChIP and library preparation were done according to [9], with modifications as described in electronic supplementary material, experimental procedures. Sequencing was performed in 100 bp reads run on an Illumina HiSeq 2500. Primer sequences used for ChIP-qPCR are provided in electronic supplementary material, experimental procedures.

### (g) LC-MS/MS (liquid chromatography–tandem mass spectrometry) of FLAG-HA-tagged KAP1

KAP1 was purified from Dignam nuclear and chromatin extracts [45] derived from K562 cells stably expressing FLAG-HA-tagged KAP1 (eKAP1), as well as from K562 cells not expressing the construct by two-step affinity chromatography [44]. Briefly, eKAP1 K562 and K562 control cells were harvested by centrifugation, washed in ice-cold full-strength PBS and resuspended in three packed cell pellet volumes of ice-cold hypotonic buffer (HB: 20 mM Tris-HCl pH 7.4, 10 mM NaCl and 1.5 mM MgCl_2_) and incubated on ice for 10 min. Cell suspensions were transferred to an ice-cooled Dounce homogenizer fitted with a B pestle, lysed with 10–15 strokes and centrifuges at 3000*g* at 4°C for 15 min to pellet nuclei. Nuclei were resuspended in one packed nuclear pellet volume of ice-cold low salt buffer (20 mM Tris-HCl pH 7.4, 0.02 M NaCl, 20% (v/v) glycerol, 0.2 mM EDTA, 1.5 mM MgCl_2_, 0.5 mM phenylmethylsulfonyl fluoride (PMSF) and 1 mM dithiothreitol (DTT)). One packed nuclear pellet volume of a high salt buffer (20 mM Tris-HCl pH 7.4, 1.2 M NaCl, 20% (v/v) glycerol, 0.2 mM EDTA, 1.5 mM MgCl_2_, 0.5 mM PMSF and 1 mM DTT) was added dropwise to the suspension. After stirring on a rotary wheel at 4 °C for 30 min to allow extraction, the suspension was centrifuged at 25 000*g* for 30 min at 4°C; the supernatant corresponded to the nuclear extract (NE). The chromatin-enriched pellet was resuspended in one packed volume of low salt buffer and one packed volume of high salt buffer, and DNA and RNA in the suspension were digested with 0.15 unit µl^−1^ benzonase (Sigma). The extraction continued on a rotary wheel at 37 °C for 15 min. The suspension was cleared by centrifugation at 25 000*g* for 30 min, and the supernatant containing the solubilized native chromatin proteins was collected. Nuclear and chromatin-soluble extracts (NE and CS, respectively) were incubated with anti-FLAG-conjugated agarose beads (A2220, Sigma) and the bound polypeptides were eluted with the FLAG peptide (Sigma) under native conditions. The affinity-purified material was then incubated with anti-HA-conjugated agarose beads (HA-agarose beads, sc-7392 AC, Santa Cruz) and eluted using HA peptide (Roche) under native conditions. Five per cent of FLAG-HA immunoaffinity-purified eKAP1 or mock immunoprecipitations from 4 l of culture was resolved on SDS-PAGE (NuPage gel, 4–12% gradient, Novex, Life Technologies) and stained with the Silverquest kit (Invitrogen). The remainder of the eluate was precipitated using the ProteoExtract Protein precipitation kit (Calbiochem) and analysed by tandem MS at the Harvard Medical School Taplin Biological Mass Spectrometry facility, Boston, MA, USA. The list of proteins with the relevant MS/MS data is provided in electronic supplementary material, table S1.

### (h) Glycerol gradient sedimentation analysis

For density gradient sedimentation, 100 μl for NE and 150 µl for CS of FLAG-purified material was loaded on a 5 ml 15–35% (v/v) glycerol gradient in buffer G (20 mM Tris-HCl pH 7.5, 150 mM NaCl, 5 mM MgCl_2_, 0.1% (v/v) Tween 20, 10 mM beta-mercaptoethanol, 0.5 mM PMSF) and centrifuged at 4°C, for 7 h at 40 000 r.p.m. in an SW 55 Ti rotor (Beckman Coulter). Fractions of 200 μl were collected from the top of the gradient, and numbered in linear, increasing order (1–25).

### (i) Bioinformatics analyses and statistics

R v. 3.1.2 (http://www.R-project.org) or GraphPad Prism v. 6.0 and 7.0 (http://www.graphpad.com) was used for statistical analyses and graphical representations of the data. H1 hESC MS/MS results [23] were visualized and handled through Scaffold (http://www.proteomesoftware.com/products/scaffold/), using the ‘quantitative value’ option, selecting hits with a *p*-value < 0.01 and a cut-off of 10 in all replicates. Proteins were further selected by the code GO0005634 for nuclear proteins. The list of proteins with the relative quantitative MS/MS data is provided in electronic supplementary material, table S1.

### (j) Western-blot antibodies

Antibodies used in these studies include: mouse anti-TRIM28 (MAB3662, Millipore), mouse anti-PCNA (NA03, Calbiochem), mouse anti-HP1Y (05-690, Millipore).

## Supplementary Material

Supplementary materials and methods, and captions for Supplementary Figures and Table

## Supplementary Material

Supplementary Table

## Supplementary Material

Supplementary Figure 1

## Supplementary Material

Supplementary Figure 2

## Supplementary Material

Supplementary Figure 3

## Supplementary Material

Supplementary Figure 4

## Supplementary Material

Supplementary Figure Legends

## Data accessibility

All next-generation sequencing data have been submitted to the NCBI Gene Expression Omnibus (GEO) (http://www.ncbi.nlm.nih.gov/geo/) database under the accession number GEO: GSE106976. Additional data used in this study correspond to the following accession numbers GEO: GSE74278 (9) and GSE87734 (44).
