# Supplementary material for: KAP1 targets actively transcribed genomic loci to exert pleomorphic effects on RNA polymerase II activity
Source: Philos Trans R Soc Lond B Biol Sci. 2020 Feb 10;375(1795):20190334. doi: 10.1098/rstb.2019.0334 (PMC7061982; doi:10.1098/rstb.2019.0334)
Supplement: Supplementary materials and methods, and captions for Supplementary Figures and Table [file rstb20190334supp1.pdf]

Supplemental Procedures

ChIP-PCR and ChIP-seq. Cells were washed with PBS, fixed for 10 minutes at 1% formaldehyde, and quenched with glycine (at 125 mM final) for 5 minutes at room temperature. Cells were washed three times with ice-cold PBS, and harvested. The pellet was lysed, resuspended in 1 mL of sonication buffer (10 mM Tris at pH 8, 200 mM NaCl, 1 mM EDTA, 0.5 mM EGTA, 0.1% NaDOC, 0.5% NLS, and protease inhibitors), transferred to TC 12x12 tubes (Covaris), and sonicated (Covaris settings: 30 minutes, 5% duty cycle, 140W, 200 cycles). Chromatin was de-crosslinked (RNAse A at 1μg/μL, 65°C overnight), purified and quantified by Nanodrop. Fragment size was assessed on a Bioanalyzer High Sensitivity chip (Agilent 2100). Immunoprecipitations were performed with 40 ug of chromatin (for KAP1), or 20 ug of chromatin (for histone modifications), with antibody-coupled magnetic beads (Dynabeads, ThermoFisher) in IP buffer (10 mM Tris at pH 8.0, 100 mM NaCl, 1 mM EDTA, 0.5 mM EGTA, 2% Triton X-100, and protease inhibitors) overnight. Chromatin was de-crosslinked (Proteinase K at 400ng/μL, 65°C overnight) and DNA purified for analysis. Antibodies used were KAP1 (Trono laboratory, rabbit polyclonal S23470), PolII CTD4H8 (Millipore, 05-623), H3K9me3 (Diagenode, C15410056), H3K4me1 (Diagenode, pAb-037-050), H3K27ac (Abcam, ab4729). ChIP samples were used for qPCR analysis by SYBER Green (Applied Biosystems) or for library preparation. Libraries of ChIP-ed DNA and the corresponding total input were performed with single-end adaptors according to (1). Sequencing runs were of 100 base-pairs (bp) reads on Illumina HiSeq 2500 (Illumina).

Primers sequences.

**Primer**	**Sequence**	**Purpose**
Actin γ	F:TGGATCAGCAAGCAGGAGTATG<br>R: CCTGCTCAGTCCATCTAGAAGCA	RT-qPCR

TBP	F:TTGACCTAAAGACCATTGCACTTC<br>R: TTCTCATGATGACTGCAGCAAA	RT-qPCR
HSPA1α	F: TTTGTGTTTGGACTCTCCCC<br>R: GCAAGGAGAAGCAGCAGAGT	RT-qPCR
HSPA1β	F: TTGACCTAAAGACCATTGCACTTC<br>R: TTGTCCATGTTAAGGTTTTGTGGTATA	RT-qPCR
SNORD104	F: CGGCGATGATGACACTCCATAC<br>R: TCACCCGGATCAGCAGTCTAAC	RT-qPCR
SNORD53	F: ATGGTTTCGCGTCTGTCTGAG<br>R: CAGCCAAGAGAAAGGTGTCATC	RT-qPCR
SNORD35a	F: GCATCAGCTTAGCCTTTG<br>R: TTCGGATGCCACAGTTAG	RT-qPCR

Bioinformatics Analyses

Genes coordinates. Genes coordinates were downloaded from BioMart 67 on Ensembl (https://www.ensembl.org/index.html) (2). The promoter region was defined taking 250 base-pairs (bp) upstream and downstream of the annotated TSS, while the gene-body comprised the rest of the gene length.

Gene Ontology Analysis. The gene ontology was performed through the PANTHER gene ontology webtool (**http://pantherdb.org**) (3). Over-representation test was performed over the background list of all annotated genes, with GO category of molecular function. Hits with Benferroni corrected p-values (p-val) lower than 1.0E-10 are reported.

Gene Features Peaks Annotation. The distribution of KAP1 binding sites over selected gene features was obtained through the R package ChIPseeker (4).

RNA-Seq. Reads were aligned to the mouse genome (mm9) with TopHat (v2.0.11) in sensitive mode (the exact parameters were: tophat -g 1 --no-novel-juncs --no-novel-indels -G $gtf --transcriptome-index $ transcriptome --b2-sensitive -o $localdir $index $reads). Gene counts were generated with HTSeq-count. Normalization for sequencing

depth and differential gene expression analysis was performed as implemented in the DESeq2 package of Bioconductor (5). P-values were corrected for multiple testing using the Benjamini-Hochberg method (6). To be considered significantly up-regulated, a gene had to have 1.5-fold increased expression and an adjusted p-value lower than 0.05. To be considered significantly down-regulated, a gene had to have 1.5-fold decreased expression and an adjusted p-value lower than 0.05.

ChIP-seq. Reads were mapped using Bowtie (7, 8) to the mm9 mouse genome assembly. Bowtie1 was used for peak-calling files, while Bowtie2 was used for coverage plots. Peaks were called using MACS (9) for transcription factors and RNA polymerase II, using the total input as control, while for histone modifications the ChIP-part module of the ChIP-seq analysis platform (http://ccg.vital-it.ch/chipseq/) was used (10).

Bigwig files. BigWigs files for the ChIP-seq experiments were generated using the Bedtools (11) genomecov program followed by the bedGraphToBigWig program from the UCSC tools (12, 13). The scaling factor option was set for each file in order to obtain a BigWig normalized to reads per 100 milion mapped reads.

Coverage plots. For PolII ChIP-seq profiles and PI establishment, coverages of the promoter region and of the gene body were defined through BamStas04 function of https://github.com/lindenb/jvarkit. The annotated gene start coordinate with a flank of 250 nucleotides in each direction was considered as the promoter region. In boxplot analyses, Mann-Whitney-Wilcoxon test was used to assess significance (NS if p-value>=0.01).

Correlation studies. Correlation analyses of ChIP-Seq data were performed with the online tool ChIP-Cor (http://ccg.vital-it.ch/chipseq/chip_cor.php) (10) using input files in BED format. Histograms were displayed as raw counts or after global normalization (as specified in the plot's y-axis).

Supplemental References

1. Rowe HM, Kapopoulou A, Corsinotti A, Fasching L, Macfarlan TS, Tarabay Y, et al. TRIM28 repression of retrotransposon-based enhancers is necessary to preserve transcriptional dynamics in embryonic stem cells. Genome research. 2013 Mar;23(3):452-61. PubMed PMID: 23233547. Pubmed Central PMCID: 3589534.
2. Yates A, Akanni W, Amode MR, Barrell D, Billis K, Carvalho-Silva D, et al. Ensembl 2016. Nucleic acids research. 2016 Jan 04;44(D1):D710-6. PubMed PMID: 26687719. Pubmed Central PMCID: 4702834.
3. Thomas PD, Campbell MJ, Kejariwal A, Mi H, Karlak B, Daverman R, et al. PANTHER: a library of protein families and subfamilies indexed by function. Genome research. 2003 Sep;13(9):2129-41. PubMed PMID: 12952881. Pubmed Central PMCID: 403709.
4. Yu G, Wang LG, He QY. ChIPseeker: an R/Bioconductor package for ChIP peak annotation, comparison and visualization. Bioinformatics. 2015 Jul 15;31(14):2382-3. PubMed PMID: 25765347.
5. Love MI, Huber W, Anders S. Moderated estimation of fold change and dispersion for RNA-seq data with DESeq2. Genome biology. 2014;15(12):550. PubMed PMID: 25516281. Pubmed Central PMCID: 4302049.
6. Benjamini Y, and Hochberg, Y. Controlling the False Discovery Rate: A Practical and Powerful Approach to Multiple Testing. Journal of the Royal Statistical Society Series B (Methodological) 1995;57:289-300.
7. Langmead B, Salzberg SL. Fast gapped-read alignment with Bowtie 2. Nature methods. 2012 Mar 04;9(4):357-9. PubMed PMID: 22388286. Pubmed Central PMCID: 3322381.
8. Langmead B, Trapnell C, Pop M, Salzberg SL. Ultrafast and memory-efficient alignment of short DNA sequences to the human genome. Genome biology. 2009;10(3):R25. PubMed PMID: 19261174. Pubmed Central PMCID: 2690996.
9. Zhang Y, Liu T, Meyer CA, Eeckhoute J, Johnson DS, Bernstein BE, et al. Model-based analysis of ChIP-Seq (MACS). Genome biology. 2008;9(9):R137. PubMed PMID: 18798982. Pubmed Central PMCID: 2592715.
10. Ambrosini G, Dreos R, Kumar S, Bucher P. The ChIP-Seq tools and web server: a resource for analyzing ChIP-seq and other types of genomic data. BMC genomics. 2016 Nov 18;17(1):938. PubMed PMID: 27863463. Pubmed Central PMCID: 5116162.
11. Quinlan AR, Hall IM. BEDTools: a flexible suite of utilities for comparing genomic features. Bioinformatics. 2010 Mar 15;26(6):841-2. PubMed PMID: 20110278. Pubmed Central PMCID: 2832824.
12. Kent WJ, Sugnet CW, Furey TS, Roskin KM, Pringle TH, Zahler AM, et al. The human genome browser at UCSC. Genome research. 2002 Jun;12(6):996-1006. PubMed PMID: 12045153. Pubmed Central PMCID: 186604.
13. Kent WJ, Zweig AS, Barber G, Hinrichs AS, Karolchik D. BigWig and BigBed: enabling browsing of large distributed datasets. Bioinformatics. 2010 Sep 01;26(17):2204-7. PubMed PMID: 20639541. Pubmed Central PMCID: 2922891.

Electronic Supplementary Material: Figures and tables (files provided separately)

Supplementary Figure 1

A. *(Left)* Fractionation profile of K562 cells untransduced (lane 1) of 3 clones transduced with pOZ-KAP1 (lanes 2,3,4). 10 μg of cytoplasmic fraction (S100) and nuclear fraction (NE) were analyzed by WB using the indicated antibodies. Clone 2 was used for subsequent studies. *(Right)* Western blot analysis with antibody targeting HA (tag of KAP1) of chromatin-soluble and nuclear extracts (CS and NE, respectively) of total and immuno-precipitated protein fractions purified from control cells (MOCK) and cells expressing the doubly-tagged form of KAP1 (KAP1). These samples were subjected to MS/MS analysis.

B. (*Left*) Western blot analysis with antibody targeting HA (one of the two tags of KAP1) of chromatin-soluble (*top*) and nuclear (*bottom*) extracts purified from cells expressing the tagged version of the protein, separated by linear glycerol gradient (15-35%) followed by ultracentrifugation. 25 fractions were collected, and uneven ones loaded on the gel. The first fraction corresponds to the lowest percentage of glycerol, while the last one to the highest. 1% of the total protein input was loaded in the first lane of the gel. (*Right*) Same settings as in the previous western blot analyses presented at the top figure panel, performed on KAP1-coimmunoprecipitated material from cells expressing the doubly-tagged form of KAP1.

C. Functional classification of proteins detected by detected in KAP1-IP MS/MS, using all annotated genes as background. Proteins co-purified (*left*) by overexpression and immunoprecipitation of the tagged version of KAP1 in nuclear and chromatin-soluble fractions of K562 cells and (*right*) by immunoprecipitation of endogenous KAP1 in H1 hESC are represented.

D. Bar plot of the peptide coverage (in %) per protein detected in the KAP1-IP MS/MS analysis performed in K562 cells. Proteins detected through a single peptide were discarded. Chromatin-soluble extracts (CS) and nuclear extracts (NE) are reported separately. KAP1 interactors previously detected in unfractionated human embryonic stem cells and K562 cells (threshold p.value <0.01) (23) are indicated with blue asterisk in this and following panel.

E. Bar plot of the total peptide count per protein detected in the KAP1-IP MS/MS analysis performed in K562 cells. Proteins detected through a single peptide were discarded. Chromatin-soluble extracts (CS) and nuclear extracts (NE) are reported separately.

Supplementary Figure 2

A. Positional correlation between KAP1 binding sites and H3K9me3-enriched loci in Hepa 1.6 cells.

B. (*Top*, *left*) Positional correlation between KAP1 binding sites and intracisternal A-particle (IAP) retrotransposons in Hepa 1.6 cells and E3 murine embryonic stem cells (mES) cells; (*top, right*) illustrative view of an IAP element locus: tracks reported are, from the top: the track of annotated genes, ChIP-seq profiles of KAP1 and of H3K9me3 in Hepa 1.6, the track of the following groups of repetitive elements: SINEs, LINEs, LTRs, and DNA repeats.

(*Bottom*, *left*) Positional correlation between KAP1 binding sites and 3' end region of KRAB zinc-finger protein (KZFP) genes in Hepa 1.6 cells and E3 murine embryonic stem cells (mES) cells; (*bottom, right*) illustrative representation of the Kzfp180 gene locus: tracks from the top: the track of annotated genes, ChIP-seq profiles of KAP1 and of H3K9me3 in Hepa 1.6, the track of the following groups of repetitive elements: SINEs, LINEs, LTRs, and DNA repeats.

* For the correlation plots, a symmetric window of 10 kilobase-pairs was considered, and the correlation was normalized by the size of each dataset.

Supplementary Figure 3

A. (*Left*) *Kap1* KD validation by RT-qPCR of Hepa 1.6 mRNA samples used for mRNA sequencing. Expression levels were normalized to small nucleolar RNA genes (SNORD35a, SNORD104, SNORD53), and the ratio between WT cells transduced with an empty sh-vector and KD cells respectively transduced with a *Kap1*-targeting sh-vector, is represented in the bar-plot. (*Right*) *Kap1* KD validation by RT-qPCR of Hepa 1.6 mRNA samples used for PolII ChIP sequencing. Expression levels were normalized to house-keeping genes transcripts *Gapdh* and *Actin β*. Besides *Kap1*, other control genes are reported in the bar plot (*Actin γ*, *Hnf4* and *Tbp)*.

B. Table reporting counts for the gene groups analyzed in Fig. 3 A-C.

C. PolII enrichment profiles over genes binned in 20 segments in WT (*left*) and *Kap1* KD (*right*) Hepa 1.6 cells. For clarity reasons, flanking regions upstream or downstream and the gene itself were labeled with different colors. Both those flanking regions measure 40% of the gene length. In each datasets, we separated genes based on the overlap of their promoter region with a KAP1 peak.

Supplementary Figure 4

A. Schematic view of the *Hspa1b* and *Hspa1a* locus displaying, in order from the top, the track of annotated genes with arrows indicating the orientation of the genes, H3K27ac and H3K4me1 ChIP-seq profiles in Hepa 1.6 cells.

B. Immuno-fluorescence analysis of (*top panels*) MEF *Kap1* KO in untreated conditions; (*middle and bottom panels*) MEF WT cells in untreated conditions and after heat-shock. Cells were stained with DAPI and an antibody targeting KAP1 or pS824 KAP1 as indicated on the left of each panel.

C. Western blot analysis of protein extracts purified from the same cells used for the experiment presented in Fig. 4B, developed with antibody targeting KAP1 and the loading

controls PCNA and CBX3. The loaded protein samples are purified from untransduced (UT) cells and cell transduced with either en empty sh-vector (WT) or a *Kap1*-targeting sh-vector (shKap1).

D. Schematic view of the inducible genes *Ier5* and *Junb* loci displaying, in order from the top: superimposed PolII ChIP-seq profiles in *Kap1* KD and WT Hepa 1.6 cells, track of annotated genes, mRNA-seq signal of *Kap1* KD and WT Hepa 1.6 cells.

Supplementary Table 1

LC-MS/MS data of the doubly-tagged version of KAP1 expressed in and purified from K562 cells, and of the endogenous protein purified from H1 hESCs (1). For the first dataset, cofactors detected in the nuclear and chromatin-soluble fractions are distinguished.
