# Supplementary material for: KAP1 targets actively transcribed genomic loci to exert pleomorphic effects on RNA polymerase II activity
Source: Philos Trans R Soc Lond B Biol Sci. 2020 Feb 10;375(1795):20190334. doi: 10.1098/rstb.2019.0334 (PMC7061982; doi:10.1098/rstb.2019.0334)
Supplement: Supplementary Figure 1 [file rstb20190334supp3.pdf]

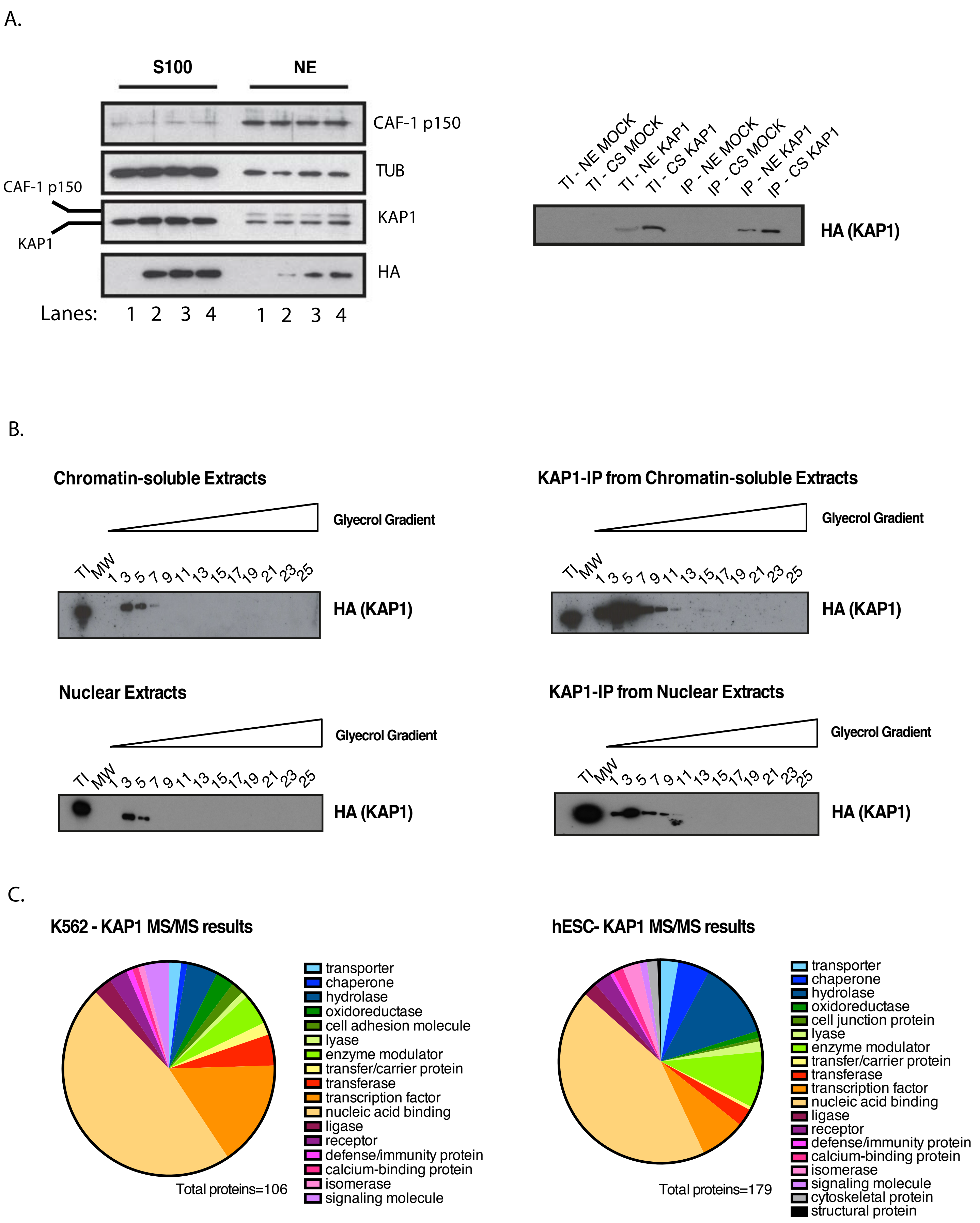
A.
S100
NE
CAF-1 p150
TUB
KAP1
HA
CAF-1 p150
KAP1
Lanes: 1 2 3 4 1 2 3 4
TI - NE MOCK
TI - CS MOCK
TI - NE KAP1
TI - CS KAP1
IP - NE MOCK
IP - CS MOCK
IP - NE KAP1
IP - CS KAP1
HA (KAP1)
B.
Chromatin-soluble Extracts
Glyecrol Gradient
TI MW 1 3 5 7 9 11 13 15 17 19 21 23 25
HA (KAP1)
KAP1-IP from Chromatin-soluble Extracts
Glyecrol Gradient
TI MW 1 3 5 7 9 11 13 15 17 19 21 23 25
HA (KAP1)
Nuclear Extracts
Glyecrol Gradient
TI MW 1 3 5 7 9 11 13 15 17 19 21 23 25
HA (KAP1)
KAP1-IP from Nuclear Extracts
Glyecrol Gradient
TI MW 1 3 5 7 9 11 13 15 17 19 21 23 25
HA (KAP1)
C.
K562 - KAP1 MS/MS results
transporter
chaperone
hydrolase
oxidoreductase
cell adhesion molecule
lyase
enzyme modulator
transfer/carrier protein
transferase
transcription factor
nucleic acid binding
ligase
receptor
defense/immunity protein
calcium-binding protein
isomerase
signaling molecule
Total proteins=106
hESC- KAP1 MS/MS results
transporter
chaperone
hydrolase
oxidoreductase
cell junction protein
lyase
enzyme modulator
transfer/carrier protein
transferase
transcription factor
nucleic acid binding
ligase
receptor
defense/immunity protein
calcium-binding protein
isomerase
signaling molecule
cytoskeletal protein
structural protein
Total proteins=179

FIGURE S1.

D.

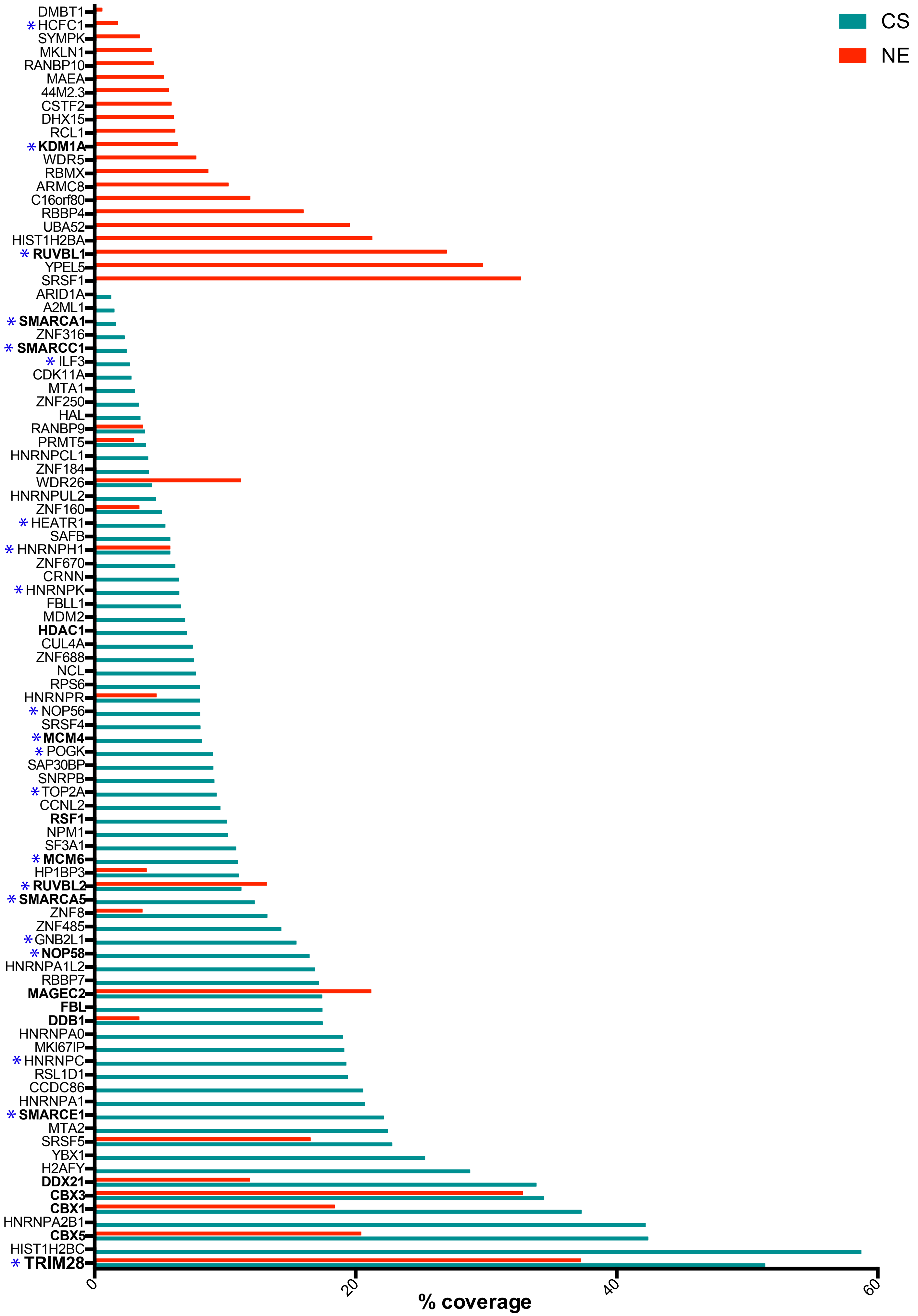

CS
NE
DMBT1
* HCFC1
SYMPK
MKLN1
RANBP10
MAEA
44M2.3
CSTF2
DHX15
RCL1
* KDM1A
WDR5
RBMX
ARMC8
C16orf80
RBBP4
UBA52
HIST1H2BA
* RUVBL1
YPEL5
SRSF1
ARID1A
A2ML1
* SMARCA1
ZNF316
* SMARCC1
* ILF3
CDK11A
MTA1
ZNF250
HAL
RANBP9
PRMT5
HNRNPCL1
ZNF184
WDR26
HNRNPUL2
ZNF160
* HEATR1
SAFB
* HNRNPH1
ZNF670
CRNN
* HNRNPK
FBLL1
MDM2
HDAC1
CUL4A
ZNF688
NCL
RPS6
HNRNPR
* NOP56
SRSF4
* MCM4
* POGK
SAP30BP
SNRPB
* TOP2A
CCNL2
RSF1
NPM1
SF3A1
* MCM6
HP1BP3
* RUVBL2
* SMARCA5
ZNF8
ZNF485
* GNB2L1
* NOP58
HNRNPA1L2
RBBP7
MAGEC2
FBL
DDB1
HNRNPA0
MKI67IP
* HNRNPC
RSL1D1
CCDC86
HNRNPA1
* SMARCE1
MTA2
SRSF5
YBX1
H2AFY
DDX21
CBX3
CBX1
HNRNPA2B1
CBX5
HIST1H2BC
* TRIM28
0
20
40
60
% coverage

FIGURE S1.

E.

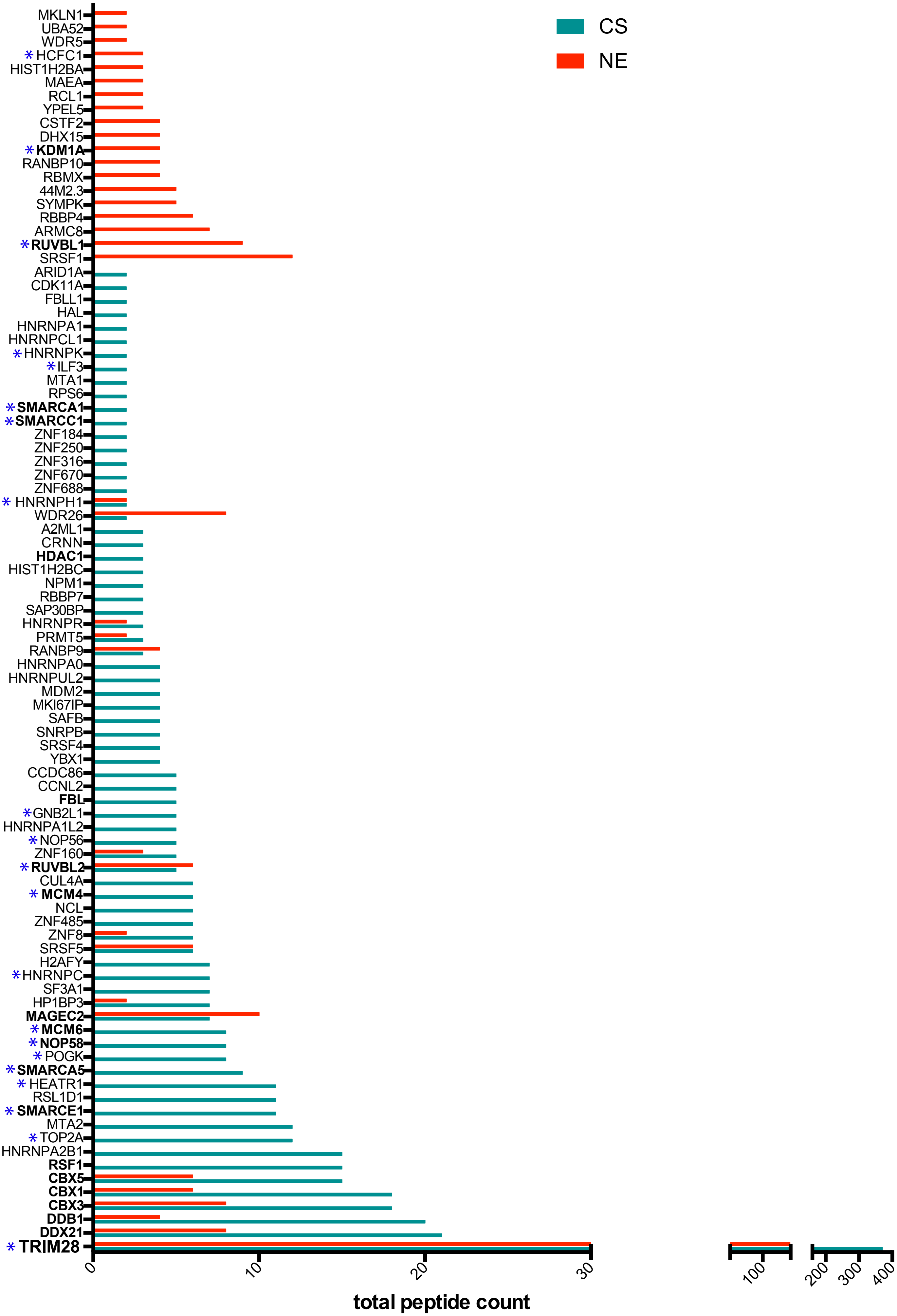
CS
NE
MKLN1
UBA52
WDR5
* HCFC1
HIST1H2BA
MAEA
RCL1
YPEL5
CSTF2
DHX15
* KDM1A
RANBP10
RBMX
44M2.3
SYMPK
RBBP4
ARMC8
* RUVBL1
SRSF1
ARID1A
CDK11A
FBLL1
HAL
HNRNPA1
HNRNPCL1
* HNRNPK
* ILF3
MTA1
RPS6
* SMARCA1
* SMARCC1
ZNF184
ZNF250
ZNF316
ZNF670
ZNF688
* HNRNPH1
WDR26
A2ML1
CRNN
HDAC1
HIST1H2BC
NPM1
RBBP7
SAP30BP
HNRNPR
PRMT5
RANBP9
HNRNPA0
HNRNPUL2
MDM2
MKI67IP
SAFB
SNRPB
SRSF4
YBX1
CCDC86
CCNL2
FBL
* GNB2L1
HNRNPA1L2
* NOP56
ZNF160
* RUVBL2
CUL4A
* MCM4
NCL
ZNF485
ZNF8
SRSF5
H2AFY
* HNRNPC
SF3A1
HP1BP3
MAGEC2
* MCM6
* NOP58
* POGK
* SMARCA5
* HEATR1
RSL1D1
* SMARCE1
MTA2
* TOP2A
HNRNPA2B1
RSF1
CBX5
CBX1
CBX3
DDB1
DDX21
* TRIM28
0
10
20
30
100
200
300
400
total peptide count
