# Supplementary figures and images for: KAP1 targets actively transcribed genomic loci to exert pleomorphic effects on RNA polymerase II activity
Source: Philos Trans R Soc Lond B Biol Sci. 2020 Feb 10;375(1795):20190334. doi: 10.1098/rstb.2019.0334 (PMC7061982; doi:10.1098/rstb.2019.0334)

**FIGURE S2.**

A.

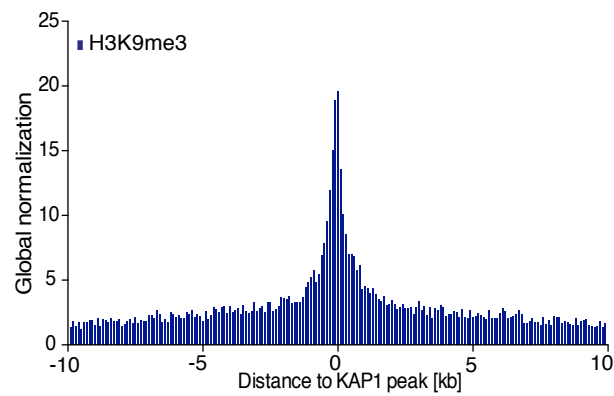

B.

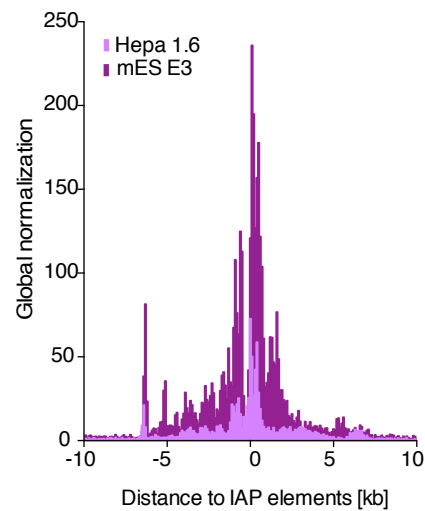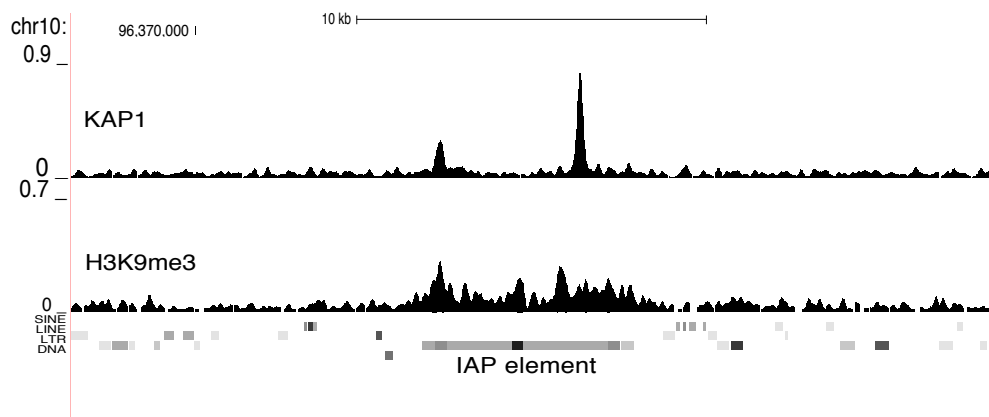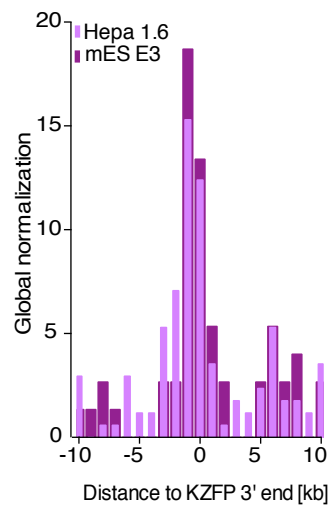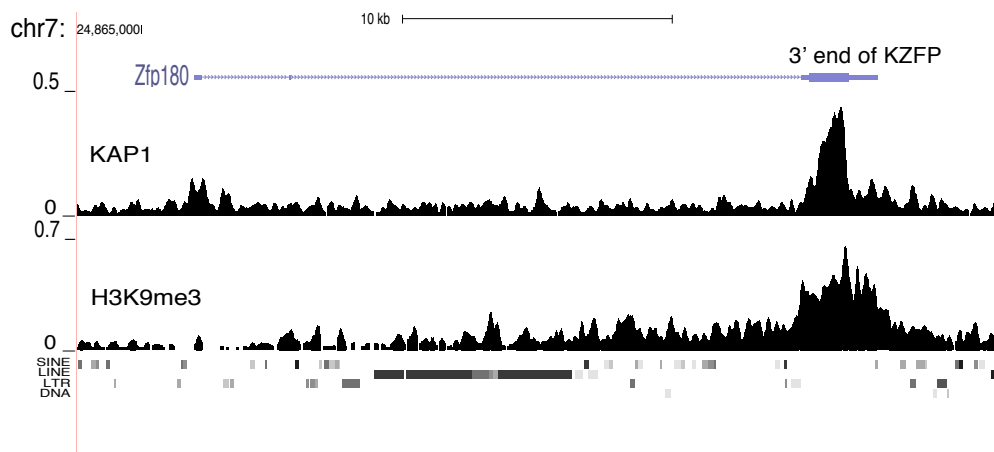

Supplement: Supplementary Figure 2 [file rstb20190334supp4.pdf]

FIGURE S4.

D.

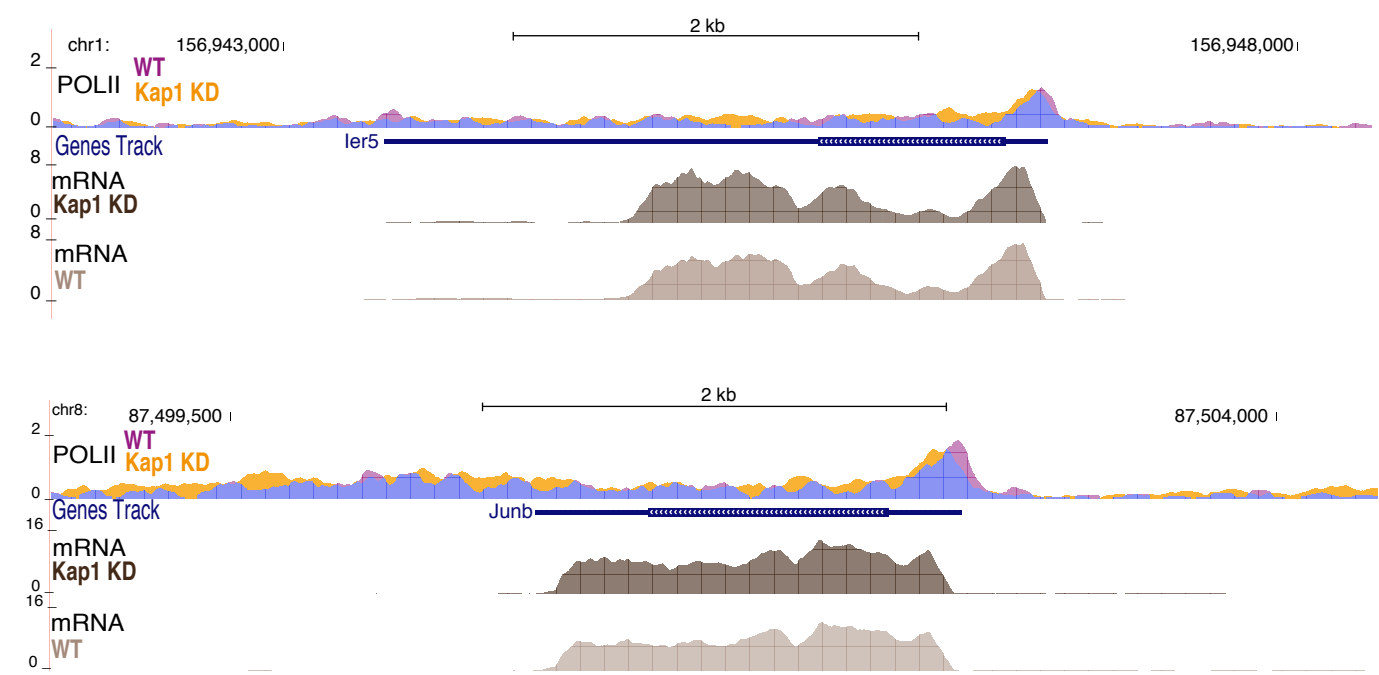

Supplement: Supplementary Figure 4 [file rstb20190334supp6.pdf]
