# Supplementary material for: KAP1 targets actively transcribed genomic loci to exert pleomorphic effects on RNA polymerase II activity
Source: Philos Trans R Soc Lond B Biol Sci. 2020 Feb 10;375(1795):20190334. doi: 10.1098/rstb.2019.0334 (PMC7061982; doi:10.1098/rstb.2019.0334)

FIGURE S3.

A.

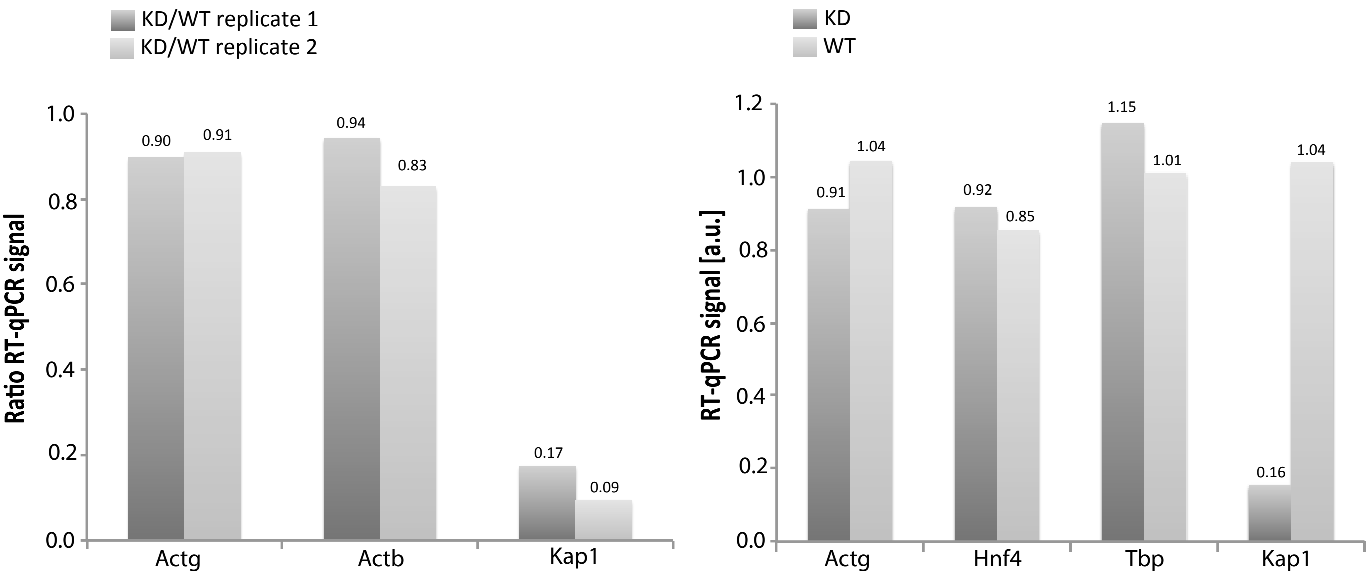

B.

Genes Dataset:	N =
gene promoters overlapping a KAP1 ChIP-Seq peak	3535
gene promoters not overlapping a KAP1 ChIP-Seq peak	19175
genes upregulated upon Kap1 KD	134
genes downregulated upon Kap1 KD	145
**Filtered genes dataset 1**	
genes filtered by PI: exclusion of Inf/NaN values	16004
gene promoters overlapping a KAP1 ChIP-Seq peak	3281
gene promoters not overlapping a KAP1 ChIP-Seq peak	12723
**Filtered genes dataset 2**	
genes filtered by PI: exclusion of Inf/NaN values, PI at basal condition >= 4	1556
promoters, KAP1 peak overlap	571
promoters, no KAP1 peak overlap	985

21 (bracket: gene promoters overlapping a KAP1 ChIP-Seq peak – genes upregulated upon Kap1 KD)
26 (bracket: gene promoters overlapping a KAP1 ChIP-Seq peak – genes downregulated upon Kap1 KD)

C.

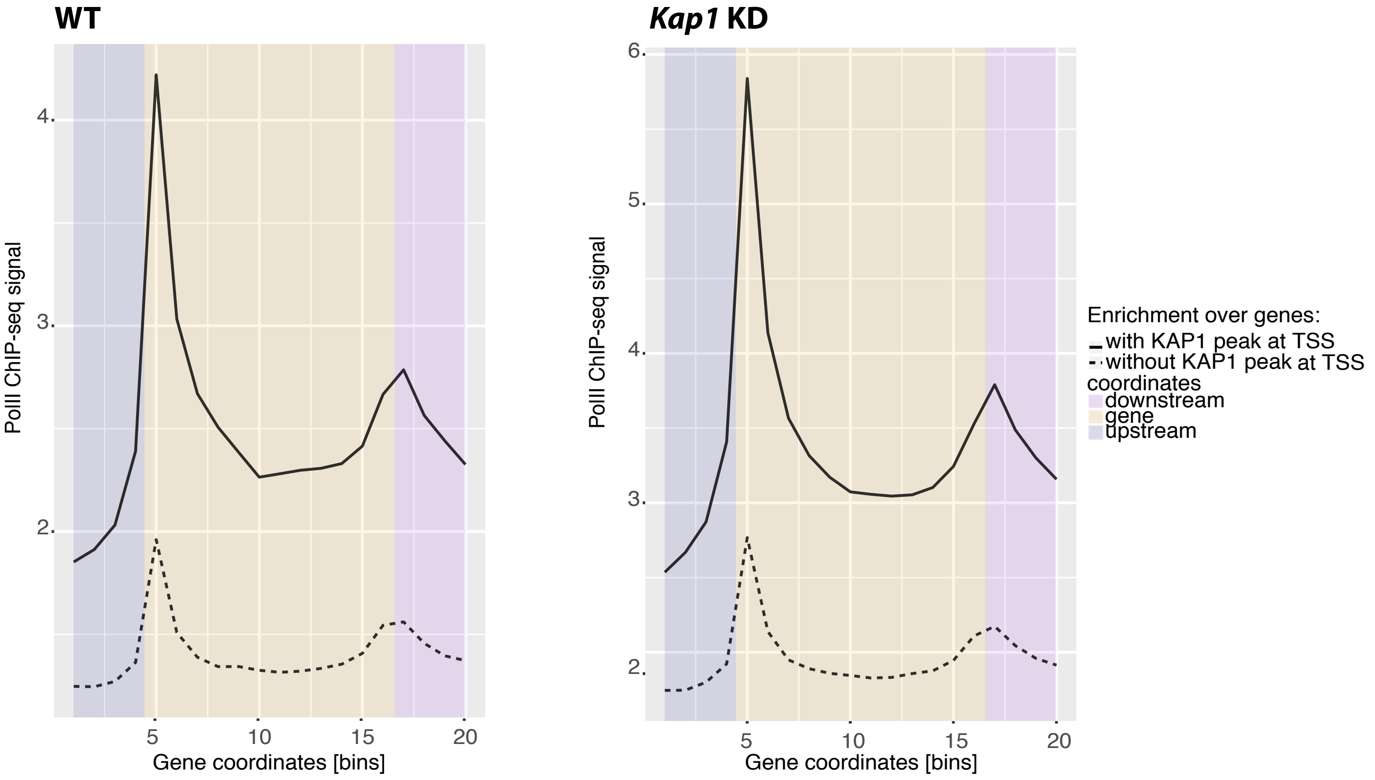

Supplement: Supplementary Figure 3 [file rstb20190334supp5.pdf]
