# Supplementary material for: KAP1 targets actively transcribed genomic loci to exert pleomorphic effects on RNA polymerase II activity
Source: Philos Trans R Soc Lond B Biol Sci. 2020 Feb 10;375(1795):20190334. doi: 10.1098/rstb.2019.0334 (PMC7061982; doi:10.1098/rstb.2019.0334)
Supplement: Supplementary Figure Legends [file rstb20190334supp7.docx]

**Electronic Supplementary Material: Titles and Captions**

**Supplementary Figure 1**

**A.** *(Left)* Fractionation profile of K562 cells untransduced (lane 1) of 3 clones transduced with pOZ-KAP1 (lanes 2,3,4). 10 µg of cytoplasmic fraction (S100) and nuclear fraction (NE) were analyzed by WB using the indicated antibodies. Clone 2 was used for subsequent studies. *(Right)* Western blot analysis with antibody targeting HA (tag of KAP1) of chromatin-soluble and nuclear extracts (CS and NE, respectively) of total and immuno-precipitated protein fractions purified from control cells (MOCK) and cells expressing the doubly-tagged form of KAP1 (KAP1). These samples were subjected to MS/MS analysis.

**B.** (*Left*) Western blot analysis with antibody targeting HA (one of the two tags of KAP1) of chromatin-soluble (*top*) and nuclear (*bottom*) extracts purified from cells expressing the tagged version of the protein, separated by linear glycerol gradient (15-35%) followed by ultracentrifugation. 25 fractions were collected, and uneven ones loaded on the gel. The first fraction corresponds to the lowest percentage of glycerol, while the last one to the highest. 1% of the total protein input was loaded in the first lane of the gel. (*Right*) Same settings as in the previous western blot analyses presented at the top figure panel, performed on KAP1-coimmunoprecipitated material from cells expressing the doubly-tagged form of KAP1.

**C.** Functional classification of proteins detected by detected in KAP1-IP MS/MS, using all annotated genes as background. Proteins co-purified (*left*) by overexpression and immunoprecipitation of the tagged version of KAP1 in nuclear and chromatin-soluble fractions of K562 cells and (*right*) by immunoprecipitation of endogenous KAP1 in H1 hESC are represented.

**D.** Bar plot of the peptide coverage (in %) per protein detected in the KAP1-IP MS/MS analysis performed in K562 cells. Proteins detected through a single peptide were discarded. Chromatin-soluble extracts (CS) and nuclear extracts (NE) are reported separately. KAP1 interactors previously detected in unfractionated human embryonic stem cells and K562 cells (threshold p.value <0.01) (23) are indicated with blue asterisk in this and following panel.

**E.** Bar plot of the total peptide count per protein detected in the KAP1-IP MS/MS analysis performed in K562 cells. Proteins detected through a single peptide were discarded. Chromatin-soluble extracts (CS) and nuclear extracts (NE) are reported separately.

**Supplementary Figure 2**

**A.** Positional correlation between KAP1 binding sites and H3K9me3-enriched loci in Hepa 1.6 cells.

**B.** (*Top*, *left*) Positional correlation between KAP1 binding sites and intracisternal A-particle (IAP) retrotransposons in Hepa 1.6 cells and E3 murine embryonic stem cells (mES) cells; (*top,* *right*) illustrative view of an IAP element locus: tracks reported are, from the top: the track of annotated genes, ChIP-seq profiles of KAP1 and of H3K9me3 in Hepa 1.6, the track of the following groups of repetitive elements: SINEs, LINEs, LTRs, and DNA repeats.

(*Bottom*, *left*) Positional correlation between KAP1 binding sites and 3’ end region of KRAB zinc-finger protein (KZFP) genes in Hepa 1.6 cells and E3 murine embryonic stem cells (mES) cells; (*bottom, right*) illustrative representation of the Kzfp180 gene locus: tracks from the top: the track of annotated genes, ChIP-seq profiles of KAP1 and of H3K9me3 in Hepa 1.6, the track of the following groups of repetitive elements: SINEs, LINEs, LTRs, and DNA repeats.

* For the correlation plots, a symmetric window of 10 kilobase-pairs was considered, and the correlation was normalized by the size of each dataset.

**Supplementary Figure 3**

**A.** (*Left*) *Kap1* KD validation by RT-qPCR of Hepa 1.6 mRNA samples used for mRNA sequencing. Expression levels were normalized to small nucleolar RNA genes (SNORD35a, SNORD104, SNORD53), and the ratio between WT cells transduced with an empty sh-vector and KD cells respectively transduced with a *Kap1*-targeting sh-vector, is represented in the bar-plot. (*Right*) *Kap1* KD validation by RT-qPCR of Hepa 1.6 mRNA samples used for PolII ChIP sequencing. Expression levels were normalized to house-keeping genes transcripts *Gapdh* and *Actin β*. Besides *Kap1*, other control genes are reported in the bar plot (*Actin 𝛾*, *Hnf4* and *Tbp)*.

**B.** Table reporting counts for the gene groups analyzed in Fig. 3 A-C.

**C.** PolII enrichment profiles over genes binned in 20 segments in WT (*left*) and *Kap1* KD (*right*) Hepa 1.6 cells. For clarity reasons, flanking regions upstream or downstream and the gene itself were labeled with different colors. Both those flanking regions measure 40% of the gene length. In each datasets, we separated genes based on the overlap of their promoter region with a KAP1 peak.

**Supplementary Figure 4**

**A.** Schematic view of the *Hspa1b* and *Hspa1a* locus displaying, in order from the top, the track of annotated genes with arrows indicating the orientation of the genes, H3K27ac and H3K4me1 ChIP-seq profiles in Hepa 1.6 cells.

**B.** Immuno-fluorescence analysis of (*top panels*) MEF *Kap1* KO in untreated conditions; (*middle and bottom panels*) MEF WT cells in untreated conditions and after heat-shock. Cells were stained with DAPI and an antibody targeting KAP1 or pS824 KAP1 as indicated on the left of each panel.

**C.** Western blot analysis of protein extracts purified from the same cells used for the experiment presented in Fig. 4B, developed with antibody targeting KAP1 and the loading controls PCNA and CBX3. The loaded protein samples are purified from untransduced (UT) cells and cell transduced with either en empty sh-vector (WT) or a *Kap1*-targeting sh-vector (shKap1).

**D.** Schematic view of the inducible genes *Ier5* and *Junb* loci displaying, in order from the top: superimposed PolII ChIP-seq profiles in *Kap1* KD and WT Hepa 1.6 cells, track of annotated genes, mRNA-seq signal of *Kap1* KD and WT Hepa 1.6 cells.

**Supplementary Table 1**

LC-MS/MS data of the doubly-tagged version of KAP1 expressed in and purified from K562 cells, and of the endogenous protein purified from H1 hESCs ([1](#_ENREF_1)). For the first dataset, cofactors detected in the nuclear and chromatin-soluble fractions are distinguished.
